# Adverse Pregnancy Outcome in Polycystic Ovarian Syndrome: A Comparative Study

**DOI:** 10.7759/cureus.25790

**Published:** 2022-06-09

**Authors:** Lipipuspa Pattnaik, Shaik A Naaz, Banya Das, Putul Dash, Manasi Pattanaik

**Affiliations:** 1 Obstetrics and Gynecology, Kalinga Institute of Medical Sciences, Bhubaneswar, IND

**Keywords:** preterm birth, sab, gdm, pregnancy outcome, pcos

## Abstract

Introduction

A paucity of data exists regarding pregnancy outcome data in women with polycystic ovarian syndrome (PCOS). Therefore, we conducted this study to compare the pregnancy outcomes of women with and without in Indian population.

Materials and methods

A total of 102 antenatal pregnant women aged between 18 and 45 years were included in this study conducted at the Department of Obstetrics and Gynecology, Pradyumna Bal Memorial Hospital, Kalinga Institute of Medical Sciences, Bhubaneswar, India. Fifty-one women had PCOS, and 51 women served as controls. We recorded patient demographic, clinical, menstrual, and pregnancy data for each group. All participants were monitored until delivery, and we recorded maternal outcomes, including spontaneous abortion, preterm birth, gestational diabetes mellitus (GDM), and pregnancy-induced hypertension (PIH). We used IBM SPSS Statistics version 20.0 for Windows (Armonk, NY: IBM Corp.) for statistical analysis and the chi-square test to analyze relationships in categorical variables.

Results

Most participants were aged between 20 and 30 years (64.7%). A high body mass index (BMI) was twice as common in women with PCOS than the control group. Most women with PCOS with pregnancy complications were overweight (62.7%) with a BMI of 25 to 29.9 kg/m^2^. A majority of women in the PCOS group (86.3%) required reproductive technology assistance, while none in the control group needed the same type of assistance. In the PCOS group, spontaneous abortions (SAB) occurred in 5.9%, GDM occurred in 17.6%, PIH in 21.6%, and preterm births in 33.3%. By contrast, the control group saw SAB occur in only 3.9%, GDM occurred in 9.8%, PIH was identical in 21.6%, and preterm births occurred in 17.6% of women without PCOS. Cesarian delivery occurred in 64.7% of women with PCOS, while only 39.2% of women without PCOS had cesarian delivery was statistically not significant.

Conclusion

We conducted this study to assess the impact of PCOS on pregnancy against pregnant women without PCOS. Pregnant women with PCOS were more likely to experience complications such as SAB, GDM, and preterm birth than pregnant women without PCOS. Therefore, pregnancies in women with PCOS are high-risk pregnancies that require frequent and timely antenatal care.

## Introduction

Polycystic ovarian syndrome (PCOS) is a common endocrine disorder in women during their reproductive year that adversely affects their fertility and reproductive health [1]. According to the Rotterdam criteria, PCOS is defined by the presence of any two of these three features: oligomenorrhea/amenorrhea, clinical or biochemical signs of hyperandrogenism, and polycystic ovaries [2]. Polycystic ovaries are defined by either the presence of ≥12 follicles measuring 2 to 9 mm or ovaries with a volume >10 ccs [3]. Due to low fertility from PCOS, affected women often seek ovulation induction or in-vitro fertilization (IVF) despite a high chance of multiple gestations and associated complications. Fertility can improve with lifestyle changes and correcting metabolic and endocrine derangements. However, very little data exist on the effect of PCOS on pregnancy in women in an Indian population. Therefore, we conducted a prospective case-control study on pregnant women in India who conceived spontaneously or by assisted reproductive technology to evaluate the pregnancy outcomes in PCOS women against those without PCOS.

## Materials and methods

This comparative prospective case-control study was performed in the Department of Obstetrics and Gynecology, Pradyumna Bal Memorial Hospital, Kalinga Institute of Medical Sciences, a tertiary care center in Bhubaneswar, India from September 2019 to September 2021. The study participants were pregnant women receiving outpatient department care. The hospital's institutional ethics committee approved the study design (approval no. 105/2019).

Pregnant women aged between 18 and 45 years were included in the study, with or without PCOS. Patients were excluded if they had anovulation due to reasons other than PCOS, were outside the age range of 18-45 years, had obesity for reasons other than PCOS, and hirsutism due to adrenal or other causes. We also excluded women with a history of diabetes mellitus, hypertension, and thyroid disorders. We also excluded women with a history of multiple pregnancies or hyperandrogenism. Finally, we excluded women receiving drug therapy. We screened all participants according to the Rotterdam criteria and assigned them to the PCOS or control group accordingly [2].

We recorded the demographic profiles of study participants. All participants received a clinical examination to record their height, weight, body mass index (BMI), blood pressure, and signs of hyperandrogenism (e.g., facial hair or acne). We noted their pregnancy history, age, parity, obstetric status, spontaneous or assisted conception, and medications. We also recorded their menstrual history in detail, including the pattern of onset, duration of bleeding, the quantity of bleeding, and other associated menstrual concerns. Study participants started antenatal checkups at approximately five to six weeks. Gestational diabetes mellitus (GDM) is defined as glucose intolerance with onset or first recognition during pregnancy. Women were screened for GDM with a 75 g oral glucose tolerance test. Blood glucose of ≥140 mg/dL after two hours of glucose administration was the threshold for diagnosing GDM. Pregnancy-induced hypertension (PIH) is defined as hypertension (blood pressure ≥140/90 mm Hg) on two or more occasions at least six hours apart with or without proteinuria (≥300 mg/24 hours) after 20 weeks of gestation. Preterm birth is defined as delivery before 37 weeks of gestation. Spontaneous abortion (SAB) is defined as spontaneous pregnancy loss before 20 weeks of gestational age. All pregnant women were monitored from first antenatal check-up (ANC) after missed period via follow-up until delivery, and we recorded maternal outcomes such as SAB, preterm birth, GDM, and PIH.

Statistical analysis

We used IBM SPSS Statistics version 20.0 for Windows (Armonk, NY: IBM Corp.) for statistical analysis and the chi-square test to analyze relationships in categorical variables.

## Results

Significantly more women with PCOS (n=22, 43.1%) gave birth at an advanced maternal age (i.e., 30 to 45 years) than women without PCOS (n=10, 19.6%, p<0.009). Overall, 40 women with PCOS (78.4%) experienced complications during pregnancy compared to 27 (52.9%) women without PCOS who experienced complications. Significantly more women with PCOS had a BMI >25 kg/m^2^ (n=32, 62.75%) than women without PCOS (n=12, 23.5%, p<0.001) as shown in Table 1.

**Table 1:** Distribution of women according to age and BMI. PCOS: polycystic ovarian syndrome

	Without PCOS, n (%)	With PCOS, n (%)	Total, n (%)	p-Value
Total	51 (100%)	51 (100%)	102 (100%)	-
Age				<0.009
≤20 years	4 (7.8%)	0 (0.0%)	4 (3.9%)	
20-30 years	37 (72.5%)	29 (56.9%)	66 (64.7%)	
30-45 years	10 (19.6%)	22 (43.1%)	32 (31.4%)	
BMI in kg/m^2^				<0.001
<18.5 kg/m^2^	4 (7.8%)	11 (21.6%)	15 (14.7%)	
18.5-24.9 kg/m^2^	32 (62.7%)	5 (9.8%)	37 (36.3%)	
25-29.9 kg/m^2^	12 (23.5%)	32 (62.7%)	44 (423.1%)	
≥30 kg/m^2^	3 (5.9%)	3 (5.9%)	6 (5.9%)	

**Figure 1:**
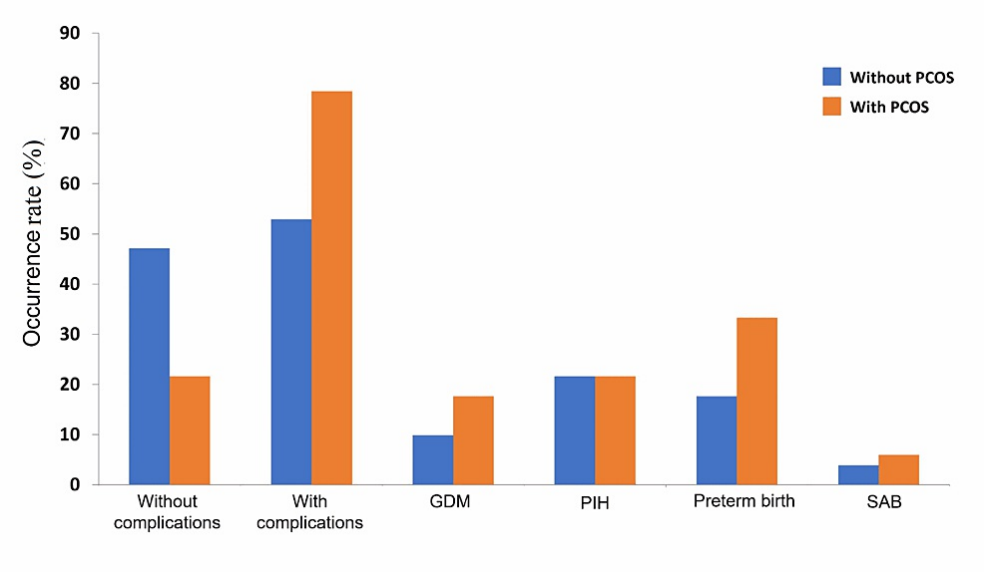
Distribution of women according to pregnancy complications. PCOS: polycystic ovarian syndrome; GDM: gestational diabetes mellitus; PIH: pregnancy-induced hypertension; SAB: spontaneous abortion

The use of assisted reproductive technology only occurred in the PCOS group (86.3%, n=44); no control group women used this technology (p<0.001) (Table 2, Figure 1).

**Table 2:** Distribution of women according to pregnancy complications. PCOS: polycystic ovarian syndrome; GDM: gestational diabetes mellitus; PIH: pregnancy-induced hypertension; SAB: spontaneous abortion

	Without PCOS, n (%)	With PCOS, n (%)	Total, n (%)	p-Value
Without complications	24 (47.1%)	11 (21.6%)	35 (34.3%)	<0.007
With complications	27 (52.9%)	40 (78.4%)	67 (65.7%)	
Complication type				
GDM	5 (9.8%)	9 (17.6%)	14 (13.7%)	-
PIH	11 (21.6%)	11 (21.6%)	22 (21.6%)	-
Preterm birth	9 (17.6%)	17 (33.3%)	26 (25.5%)	-
SAB	2 (3.9%)	3 (5.9%)	5 (4.9%)	-

Women in the PCOS group had a greater incidence of GDM (n=9, 17.6%), preterm birth (n=17, 33.3%), and SAB than women in the control group, but the incidence of PIH was identical in both groups (Table 3). Cesarean delivery was more common for women in the PCOS group (64.7%) than the control group (39.2%) (Table 4).

**Table 3:** Distribution of women according to method of conception. PCOS: polycystic ovarian syndrome; IUI: intrauterine insemination; IVF: in-vitro fertilization

Method of conception	Without PCOS, n (%)	With PCOS, n (%)	Total, n (%)	p-Value
Spontaneous	51 (100%)	7 (13.7%)	58 (56.9%)	<0.001
Assisted conception	0 (0.0%)	44 (86.3%)	44 (43.1%)	
Assisted conception type				
IUI	0 (0.0%)	4 (7.8%)	4 (3.9%)	-
IVF	0 (0.0%)	14 (27.5%)	14 (13.7%)	-
Ovulation induction	0 (0.0%)	26 (51%)	26 (25.5%)	-

**Table 4:** Distribution of women according to mode of delivery. PCOS: polycystic ovarian syndrome; SAB: spontaneous abortion

Mode of delivery	Without PCOS, n (%)	With PCOS, n (%)	Total, n (%)
Vaginal delivery	29 (56.9%)	15 (29.4%)	44 (43.1%)
Cesarean delivery	20 (39.2%)	33 (64.7%)	53 (52.0%)
SAB	2 (3.9%)	3 (5.9%)	5 (4.9%)

## Discussion

This was a hospital-based prospective comparative study to compare pregnancy outcomes in women with PCOS against women without PCOS. Delivery at an advanced age was more common in women with PCOS than women without PCOS because those women experienced chronic anovulation and delayed conception. The mean age of women with PCOS was 26.8 years (range: 21 to 44 years), similar to the mean age reported in other studies [4-6]. Also, most women with PCOS relied on assisted reproductive technology while no women in the control group did so. This was similar to an Australian study in women with PCOS that reported higher proportions of PCOS patients conceived after in-vitro fertilization than those without PCOS [4].

We saw a much higher prevalence of BMI >25 kg/m^2^ in the PCOS group than the control group, which aligned with the findings reported by De Frène et al. [7]. Their retrospective cohort study saw a higher mean BMI (30.8 kg/m^2^) in women with PCOS than in women without PCOS [7]. GDM was more common among the PCOS group than the control, supporting a meta-analysis that reported women with PCOS had a significantly increased risk of GDM [8]. In our study, the percentage of PIH was the same in both groups. The increased risk of miscarriage among women with PCOS compared to controls in our study was mirrored by the results of a large Australian study that showed higher rates of miscarriage associated with PCOS [9]. Naver et al. reported that preterm births were more common in women with PCOS than without, which aligned with our results [10]. Also, several meta-analyses described a 1.3 to 3.9-fold increased risk of preterm delivery in pregnant women with PCOS [11]. Cesarean section delivery was more common among women with PCOS in our study. These results align with a meta-analysis that concluded PCOS was associated with a significantly higher risk of cesarean delivery than women without PCOS [12].

Because of small sample size of our study, the results cannot be applied to the whole population. Moreover, there are different phenotypes in PCOS where the adverse effects may be different. We have not considered the different phenotypes which was a limitation of our study.

## Conclusions

This study compared pregnancy outcomes of women with PCOS against those without PCOS. Pregnant women with PCOS were more likely to experience complications such as SAB, GDM, and preterm birth which were statistically significant than pregnant women without PCOS. Therefore, PCOS pregnancies are high-risk pregnancies that require frequent and timely antenatal care.
